# Cardiometabolic comorbidities in autosomal dominant polycystic kidney disease: a 16-year retrospective cohort study

**DOI:** 10.1186/s12882-023-03382-0

**Published:** 2023-11-09

**Authors:** Li-Chi Chen, Yi-Chi Chu, Tzongshi Lu, Hugo Y.-H. Lin, Ta-Chien Chan

**Affiliations:** 1 Research Center for Humanities and Social Sciences, Academia Sinica, 128 Academia Road, Section 2, Nankang, Taipei 115 Taiwan; 2 Harvard T.H. Chan School of Public Health, Harvard University, Boston, MA USA; 3 Renal Division, Brigham and Women’s Hospital, Harvard Medical School, Boston, MA USA; 4 Department of Internal Medicine, Kaohsiung Municipal Ta-Tung Hospital, No.68, Jhonghua 3rd Road, Cianjin, Kaohsiung 807 Taiwan; 5 Division of Nephrology, Department of Internal Medicine, Kaohsiung Medical University Hospital, Kaohsiung Medical University, Kaohsiung, Taiwan; 6 Department of Medicine, College of Medicine, Kaohsiung Medical University, Kaohsiung, Taiwan; 7 Institute of Public Health, School of Medicine, National Yang Ming Chiao Tung University, Taipei, Taiwan

**Keywords:** ADPKD, Cardiometabolic comorbidities, ESRD, Renal replacement therapy, All-cause mortality

## Abstract

**Background:**

Autosomal-dominant polycystic kidney disease (ADPKD) is the most prevalent hereditary kidney disease and the fourth leading cause of end-stage renal disease (ESRD) requiring renal replacement therapy (RRT). Nevertheless, there is a paucity of epidemiological research examining the risk factors and survival on RRT for ADPKD. Thus, we aimed to investigate the cumulative effects of cardiometabolic comorbidities, including hypertension (HTN), type 2 diabetes mellitus (DM), and dyslipidemia (DLP) to clinical outcomes in ADPKD.

**Methods:**

We identified 6,142 patients with ADPKD aged ≥ 20 years from 2000 to 2015 using a nationwide population-based database. HTN, DM, and DLP diagnoses before or at the time of ADPKD diagnosis and different combinations of the three diagnoses were used as the predictors for the outcomes. Survival analyses were used to estimate the adjusted mortality risk from cardiometabolic comorbidities and the risk for renal survival.

**Results:**

Patients with ADPKD who developed ESRD had the higher all-cause mortality (HR, 5.14; [95% CI: 3.88–6.80]). Patients with all three of the diseases had a significantly higher risk of entering ESRD (HR:4.15, [95% CI:3.27–5.27]), followed by those with HTN and DM (HR:3.62, [95% CI:2.82–4.65]), HTN and DLP (HR:3.54, [95% CI:2.91–4.31]), and HTN alone (HR:3.10, [95% CI:2.62–3.66]) compared with those without any three cardiometabolic comorbidities.

**Conclusions:**

Our study discovered the cumulative effect of HTN, DM, and DLP on the risk of developing ESRD, which reinforces the urgency of proactive prevention of cardiometabolic comorbidities to improve renal outcomes and overall survival in ADPKD patients.

**Supplementary Information:**

The online version contains supplementary material available at 10.1186/s12882-023-03382-0.

## Background

Autosomal-dominant polycystic kidney disease (ADPKD) is the most prevalent hereditary kidney disease [1] (1:500 to 1:1000) and the fourth most common cause of the end-stage renal disease (ESRD) [2], which affects over 12 million individuals worldwide [3]. It is genetically heterogeneous, with two genes identified: *PKD1* (in 78% of disease pedigrees) on chromosome 16 (16p13.3) and *PKD2* (in 15% of disease pedigrees) on chromosome 4 (4q21) [4], encoding polycystin-1 (PC1) and polycystin-2 (PC2), respectively [5]. Mutations in either plasma membrane-spanning polycystins disrupt intracellular signaling pathways and lead to cystogenesis [6]. ADPKD is characterized by the development and progressive expansion of multiple bilateral cysts throughout the kidneys resulting in renal dysfunction. Approximately half of the patients with ADPKD develop ESRD and require renal replacement therapy (RRT) [6]. However, there is a huge variation in renal dysfunction outcomes, ranging from severe neonatal-onset ADPKD to late onset of renal dysfunction after 75 years old [7].

A large body of literature has concentrated on epidemiological evidence of risk factors for ADPKD progression including genetic mutations (*PKD1* and *PKD2*) [8], large kidney size (height-adjusted total kidney volume, htTKV) [9], early onset of hypertension [10], early and recurrent hematuria [11], and sex [12]. Furthermore, a U.S. retrospective cohort study of 22 patients with diabetes and ADPKD showed that comorbid type 2 diabetes mellitus (DM) and ADPKD were associated with larger renal volumes, earlier age at diagnosis of hypertension, and higher mortality than those with ADPKD alone [13]. However, the potential mechanisms and comprehensive evaluations of the leading risk factors for cardiometabolic comorbidities, including hypertension (HTN), DM, and dyslipidemia (DLP), in ADPKD progression remain unexplored [14].

Since there is a lack of effective treatments for ADPKD, there is an urgent need to explore potential therapeutic agents. Although tolvaptan was approved by the U.S. Food and Drug Administration (FDA) for ADPKD in 2018 [15, 16], it cannot be administered to all patients with ADPKD, particularly those with hepatic impairments. Therefore, our study assessed the drug history of metformin because it has been reported to have therapeutic potential for treating ADPKD by slowing the estimated glomerular filtration rate (eGFR) decline and causing a smaller increase in htTKV [17, 18]. We also investigated the non-steroidal anti-inflammatory drugs (NSAIDs), which were known for the nephrotoxicity if chronically used for pain management in ADPKD patients [19], to provide data for meticulous evaluation of nephrotoxic agent prescriptions.

The aim of this study was to use Taiwan’s National Health Insurance Research Database (NHIRD) with over 28 million participants to conduct a 16-year population-based cohort study to assess the predictive value of comorbidities and drug history as well as to develop models to estimate renal outcomes of cardiometabolic comorbidities (HTN, DM, DLP) and overall survival in the ADPKD population.

## Materials and methods

### Data source

Taiwan’s NHIRD derives from the claims data of the NHI program, which is a single-payer mandatory enrollment healthcare system with a coverage rate of up to 99.99% of Taiwan’s 23.26 million population [20]. The NHIRD exemplifies a population-level data source where researchers have access to robust information, including details of inpatient and ambulatory care, prescriptions dispensed at pharmacies, and health service utilization of medical facilities [21]. Furthermore, the NHIRD is linked to other healthcare databases, such as the Taiwan Cancer Registry and Causes of Death dataset, with individual personal identification numbers (PINs) that provide comprehensive patient-level information for public health research.

### Study population and design

**Fig. 1:**
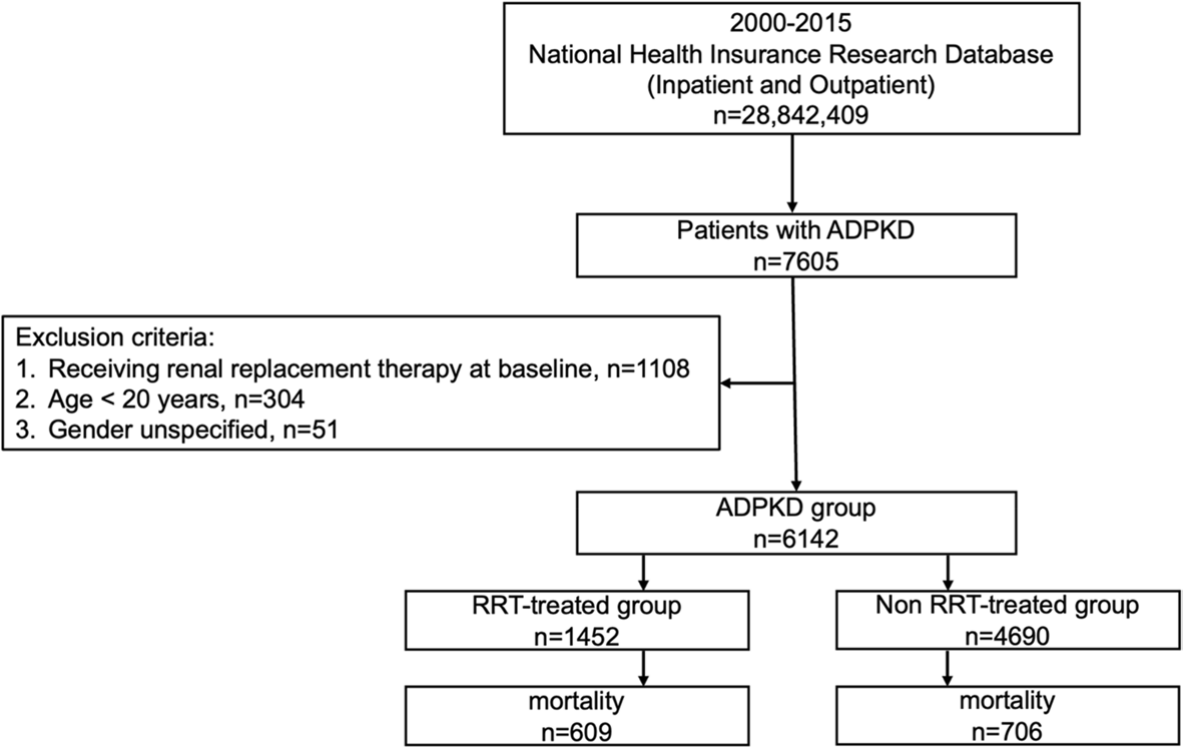
Flowchart of participant selection

We conducted a nationwide, retrospective, longitudinal cohort study using data from the 2000–2015 NHIRD. Diagnoses were coded based on the International Classification of Diseases, Ninth Revision, Clinical Modification (ICD-9-CM). Among the 28,842,409 participants during our 16-year study period, 7,605 patients were identified as having ADPKD (ICD-9-CM code: 753.13). A total of 1,463 patients with ADPKD were excluded for the following reasons: RRT prior to ADPKD diagnosis (*n* = 1,108), age less than 20-year-old (*n* = 304), and unspecified sex (*n* = 51). Altogether, 6,142 eligible participants were included in the analysis and stratified by RRT status. Approximately 1,451 patients were treated with RRT after the diagnosis of ADPKD during the study period and 4,691 were not. In addition, the mortalities in the corresponding groups were identified by linking with the causes of death dataset. A flowchart of the study participants is shown in Fig. 1.

### Predictor variables and covariates

The predictors evaluated in this study were comorbidities and drug histories at baseline. Pre-existing comorbidities for each participant were estimated as HTN (ICD-9-CM codes 401–405), DLP (ICD-9-CM code 272), and DM (ICD-9-CM code 250) (Table S1). Concomitant HTN, DM, and DLP were confirmed by at least two records of outpatient visits within 365 calendar days or by one diagnosis upon admission before or at the time of ADPKD diagnosis during the study period. Because of the high correlation between HTN, DM, and DLP in patients with ADPKD (Table S2), we addressed possible multicollinearity by classifying comorbid HTN, DM, and DLP into the following eight combinations: HTN (-) DLP (-) DM (-), HTN ( +) DLP (-) DM (-), HTN (-) DLP (-) DM ( +), HTN (-) DLP ( +) DM (-), HTN ( +) DLP ( +) DM (-), HTN ( +) DLP (-) DM ( +), HTN (-) DLP ( +) DM ( +), HTN ( +) DLP ( +) DM ( +), where ( +) represents the presence of a specific comorbidity and (-) represents the absence.

Drug history was defined as the prescription of at least 30 dispensed units of specific medications according to anatomical therapeutic chemical (ATC) codes within 365 calendar days prior to or at the time of ADPKD diagnosis. In this study, we assessed the use of metformin (ATC code A10BA02) and NSAIDs (ATC code M01A; Table S3). We identified participants who underwent RRT modalities, including hemodialysis, peritoneal dialysis, other dialysis, and renal transplantation (The definition is listed in Table S4). The index date of the RRT incidence was defined as the first day of the participant’s first dialysis session.

The covariates assessed in the study included demographic factors, including sex and age at the first diagnosis of ADPKD. The participants were categorized into three age groups: 20–39, 40–59, and ≥ 60. Individuals with incomplete information on any of the variables were excluded from the model analyses. None of the variables had more than 5% missing data.

### Statistical analysis

The baseline characteristics of the participants were summarized using descriptive statistics. Furthermore, we compared the distribution of demographic factors and the rate of comorbidities and drug history between the RRT-treated and non-RRT-treated groups using the chi-square test.

Primary statistical analysis was performed on all study participants with the endpoint of all-cause mortality, whereas secondary analysis was conducted on the RRT-treated group with the endpoint of RRT.

Hazard ratios (HRs) and two-sided 95% confidence intervals (CIs) for all-cause mortality in the primary analysis were calculated using Cox proportional hazards models, with RRT as a time-dependent covariate adjusted for sex and age. Moreover, the effects of the combination of HTN, DM, and DLP on all-cause mortality across RRT-treated and non-RRT-treated status were explored through adding multiplicative terms in the Cox proportional hazards regression model.

Using univariate and multivariate Cox proportional analyses, the HRs and CIs of the secondary analysis were computed to evaluate the significance of baseline variables regarding renal survival, which was defined as the time to RRT initiation. Moreover, we utilized Kaplan–Meier curves to compare renal outcomes among different subgroups and conducted a log-rank test to examine differences between subgroups.

Furthermore, A competing risk (Fine and Gray) model was fitted to estimate subdistribution hazard ratios (SHR) for mortality to assess the effects of RRT and factors associated with initiation of RRT, including the combinations of HTN, DM, and DLP.

The follow-up period for each patient was calculated as the difference between the dates of ADPKD diagnosis, RRT, and death. Data were right-censored if the event (RRT or death) did not occur within the study period. The censored date was December 31, 2015. The statistical significance level was set at *p*-value < 0.05. Analyses were conducted using the SAS software (version 9.4; SAS Institute, Cary, NC, USA) and R software, version 3.6.3 (R Core Team, Vienna, Austria).

### Ethics statement

The study was approved by the Institutional Review Board (IRB) on Biomedical Science Research, Academia Sinica (AS-IRB-BM-19026). Individually identifying data were removed and remained anonymous during the entire study. This study was performed in accordance with the declaration of Helsinki and followed by the approved protocol.

## Results

### Baseline characteristics of patients with ADPKD

We included 6,142 patients with ADPKD (Table 1). In this cohort, 1,452 (23.6%) commenced RRT during a median follow-up of 64.69 months, whereas 4,690 (76.4%) did not. The mean age at initiation of RRT was 59.2 ± 13.4 months. There were significant higher proportions of middle-aged (40–59) and old-aged (≥ 60 years) adults in the RRT-treated group (50.3% and 38.2%, respectively) than in the non-RRT-treated group (43.1% and 26.0%, respectively). The percentage of male patients was significantly greater in both groups. The comorbidities of HTN, DM, and DLP were remarkably higher in the RRT-treated group than in the non-RRT-treated group (*p*-values < 0.05). Patients with a history of NSAIDs use comprised a larger percentage of the non-RRT-treated group (6.93%) than in the RRT-treated group (3.31%), whereas there was no significant difference in the proportion of metformin users between the RRT-treated and non-RRT-treated groups. During a median follow-up of 71.47 months, 1,315 deaths were reported, 609 in the RRT-treated and 706 in the non-RRT-treated groups. The RRT-treated group had a significantly higher mortality rate than the non-RRT-treated group (3.59/1000 person-months v.s. 1.79 /1000 person-months, *p* < 0.001).

**Table 1:** Baseline characteristics and mortality at follow-up of ADPKD patients stratified by RRT status

	**ADPKD Group** **Counts (%)/** **Mean ± SD** **(median (p5, p95))**	**RRT-treated** **Counts (%)/** **Mean ± SD** **(median (p5, p95))**	**Non RRT-treated** **Counts (%)/** **Mean ± SD** **(median (p5, p95))**	***P*-value**
N	6142	1452 (23.6)	4690 (76.4)	
**Sex**				< .001
Male	3169 (51.6)	806 (55.5)	2363 (50.4)	
Female	2973 (48.4)	646 (44.5)	2327 (49.6)	
**Age**				< .001
20—39	1617 (26.3)	166 (11.4)	1451 (30.9)	
40—59	2752 (44.8)	731 (50.3)	2021 (43.1)	
≥ 60	1773 (28.9)	555 (38.2)	1218 (26.0)	
**Age (years) at initiation of RRT**	**-**	59.2 ± 13.4 58 (39, 82)	-	
**Comorbidities**				
Hypertension (HTN)	3951 (64.3)	1245 (85.7)	2706 (57.7)	< .001
Dyslipidemia (DLP)	1476 (24.0)	417 (28.7)	1059 (22.6)	< .001
Diabetes Mellitus (DM)	786 (12.8)	252 (17.4)	534 (11.4)	< .001
**Combinations of HTN, DLP, and DM**				< .001
HTN ( +) DLP (-) DM (-)	2372 (38.6)	737 (50.8)	1635 (34.9)	
**HTN (-) DLP ( +) DM (-)**	143 (2.33)	9 (0.62)	134 (2.86)	
**HTN (-) DLP (-) DM ( +)**	65 (1.06)	15 (1.03)	50 (1.07)	
HTN ( +) DLP ( +) DM (-)	891 (14.5)	274 (18.9)	617 (13.2)	
HTN ( +) DLP (-) DM ( +)	279 (4.54)	103 (7.09)	176 (3.75)	
HTN (-) DLP ( +) DM ( +)	33 (0.54)	3 (0.21)	30 (0.64)	
HTN ( +) DLP ( +) DM ( +)	409 (6.66)	131 (9.02)	278 (5.93)	
HTN (-) DLP (-) DM (-)	1950 (31.8)	180 (12.4)	1770 (37.7)	
**Drug History**				
**Metformin**	70 (1.14)	11 (0.76)	59 (1.26)	0.117
**NSAIDs**	373 (6.07)	48 (3.31)	325 (6.93)	< .001
**Duration of follow-up** (months)				
From ADPKD diagnosis to RRT or censor date^a^	73.82 ± 54.02 64.69 (3.32, 171.99)	40.94 ± 40.59 26.84 (0.99, 129.58)	84.00 ± 53.60 79.41 (6.74, 176.18)	< .001
From ADPKD diagnosis to death or censor date^a^	77.97 ± 52.73 71.47 (5.22, 173.0)	91.85 ± 52.11 89.54 (12.04, 180.5)	73.67 ± 52.18 65.38 (4.14, 169.5)	< .001
From RRT to death or censor date^a^	-	50.91 ± 44.45 39.70 (1.08, 138.1)	-	
**Death**	1315 (21.4)	609 (41.9)	706 (15.1)	
**Mortality Rate**^b^	2.33	3.59	1.79	

Continuous variables are presented as mean ± SD (median (p5, p95)); category variables are expressed as a count (%)

^a^Data were right-censored if the event (RRT or death) did not occur within the study period. The censor date was December 31, 2015

^b^Mortality rate is calculated as deaths/1000 person-months

### All-cause mortality of ADPKD patients

**Fig. 2:**
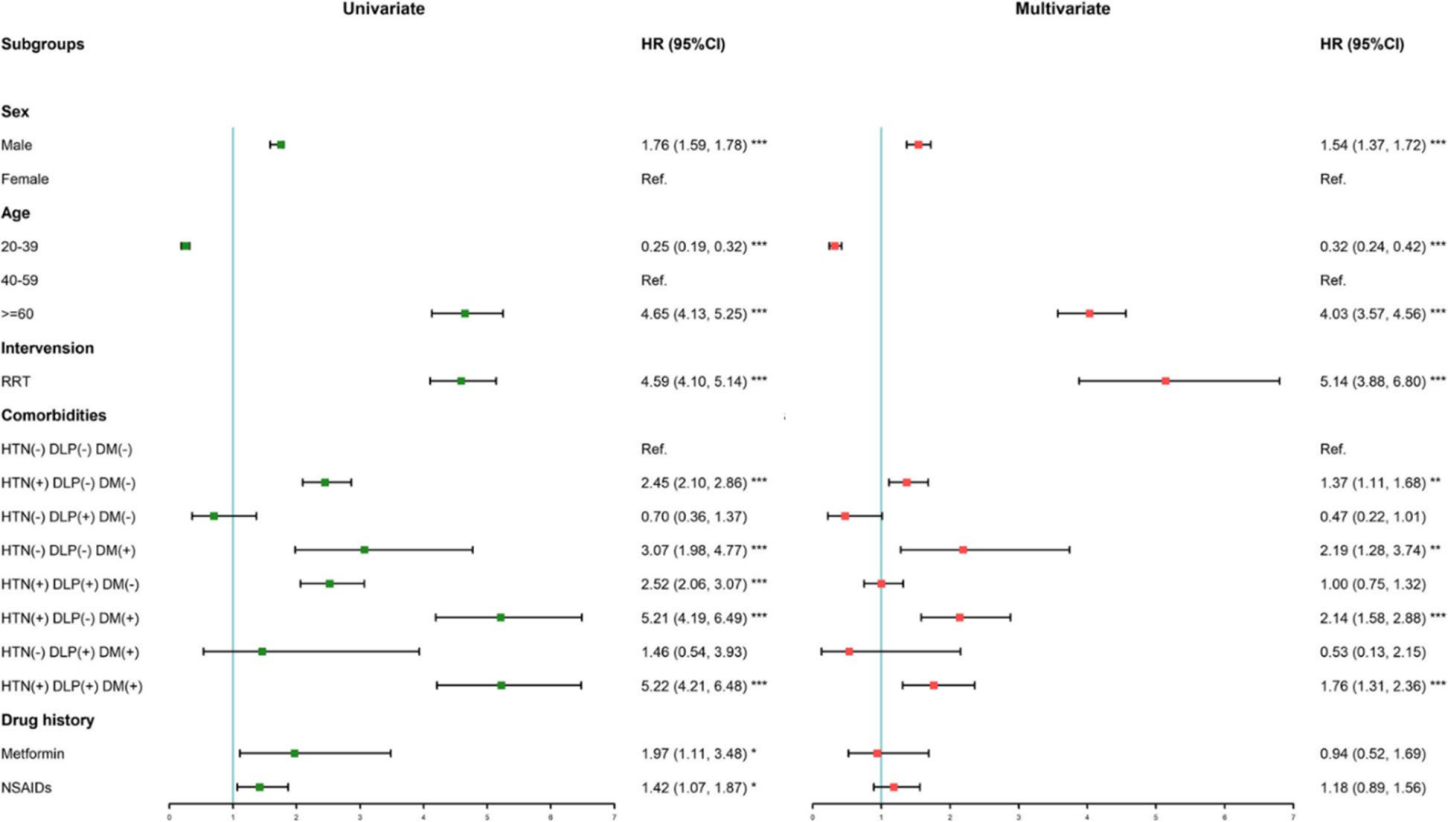
Unadjusted univariate and adjusted multivariate hazard ratios for the all-cause mortality in ADPKD patients. (*p < 0.05, **p < 0.01, ***p < 0.001). The green box denotes the point estimate of univariate hazard ratio, the pink box denotes the point estimate of multivariate hazard ratio, and the horizontal line denotes the 95% confidence interval. Multivariate hazard ratios are adjusted for age at diagnosis and sex

To investigate the impacts of clinical comorbidities of all-cause mortality in ADPKD, we examined the risk of death by using the Cox regression model with RRT as a time-dependent covariate. The mortality significantly increased with age (Fig. 2). And male sex (HR,1.54; [95% CI:1.37–1.72]) was identified as an independent risk factor. Patients receiving RRT had the worst overall survival (HR: 5.14, [95% CI: 3.88–6.80]). Higher all-cause mortalities were observed in patients with concomitant diagnoses of HTN alone (HR: 1.37, [95% CI: 1.11–1.68]), DM alone (HR: 2.19, [95% CI: 1.28–3.74]), both DM and HTN (HR: 2.14, [95% CI: 1.58–2.88]), and the combination of HTN, DM, and DLP (HR: 1.76, [95% CI: 1.31–2.36]). The analysis of consideration of the interaction effect between RRT and the combination of HTN, DM, and DLP were shown in Table S5. RRT showed effect modification of overall survival in the groups of patients with HTN alone (HR:0.54, [95% CI:0.39, 0.75]), comorbid HTN and DM (HR:0.44, [95% CI: 0.28, 0.69]), and comorbid HTN, DM, and DLP (HR:0.57, [95% CI: 0.37, 0.88]). Neither a history of metformin (HR: 0.94, [95% CI: 0.52, 1.69]) nor NSAIDs use (HR: 1.18, [95% CI: 0.89, 1.56]) was found to be a risk factor for all-cause mortality.

Fine and Gray model for all-cause mortality accounting for competing risk with RRT (Figure S1) displayed strengthened associations between all-cause mortality and groups with HTN alone (HR:3.06, [95% CI:2.58, 3.64]), comorbid HTN and DLP (HR:3.40, [95% CI:2.80, 4.14]), comorbid HTN and DM (HR:3.58, [95% CI:2.78, 4.61]), and comorbid HTN, DM, and DLP (HR:3.81, [95% CI:3.00, 4.83]) after adjustment for sex and age. The cumulative risks of comorbid HTN, DM, and DLP were shown after accounting for a competing risk with RRT.

### Risk of renal replacement therapy of ADPKD patients

**Fig. 3:**
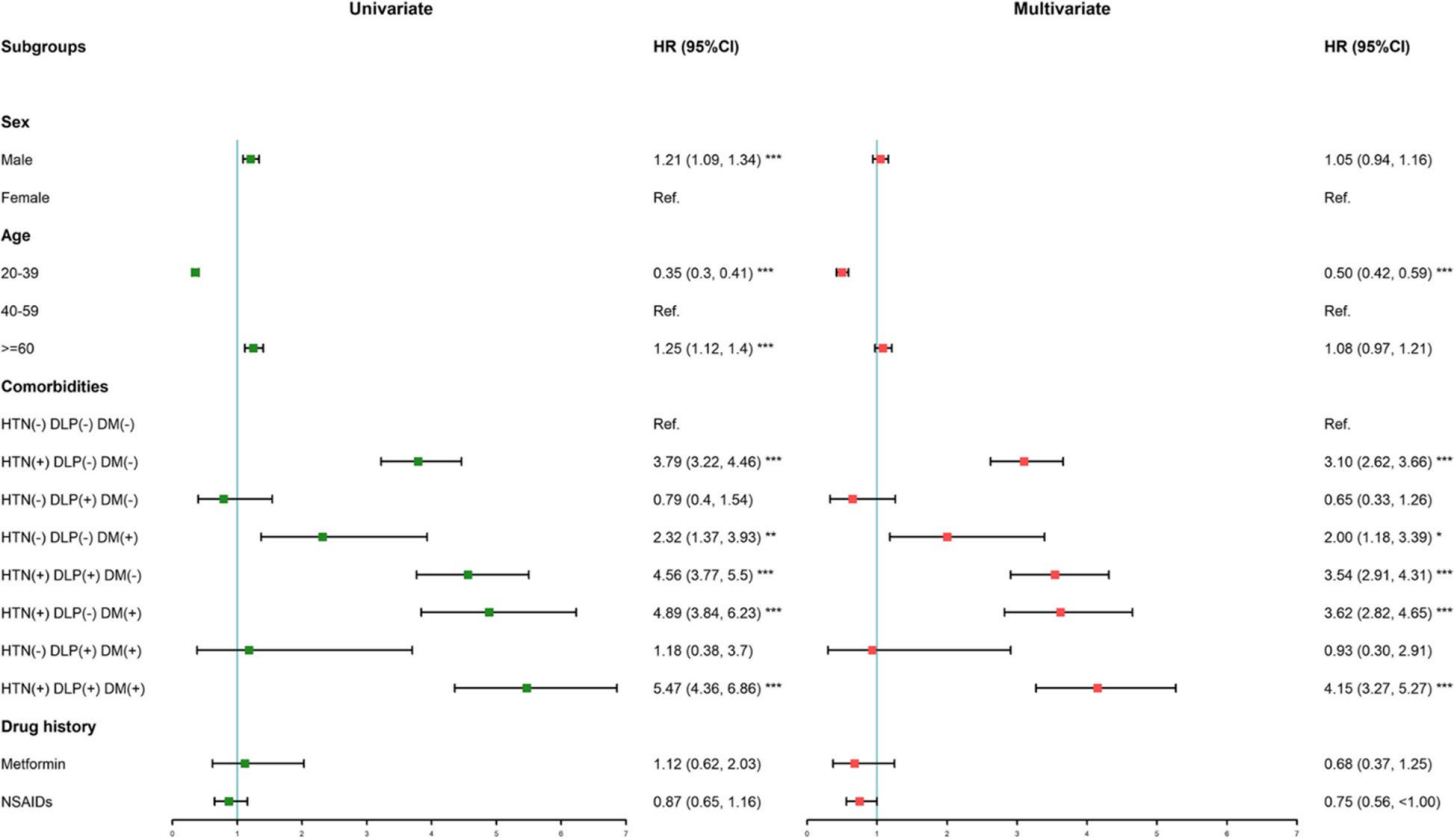
Unadjusted univariate and adjusted multivariate hazard ratios for the risk of renal replacement therapy in ADPKD patients. (*p < 0.05, **p < 0.01, ***p < 0.001). The green box denotes the point estimate of univariate hazard ratio, the pink box denotes the point estimate of multivariate hazard ratio, and the horizontal line denotes the 95% confidence interval. Multivariate hazard ratios are adjusted for age at diagnosis and sex

To examine the risk of RRT in patients with ADPKD, we analyzed and identified male sex, older age (≥ 60 years), HTN alone, DM alone, comorbid HTN and DLP, comorbid HTN and DM, and comorbid HTN, DM, and DLP as independent risk factors for RRT by using univariate Cox proportional hazard model (Fig. 3). In contrast, younger adult (20–39) had better renal outcomes. After adjusting for sex and age in the multivariate model, the cumulative effects of comorbid HTN, DM, and DLP were discovered. Patients diagnosed with all three comorbidities had the significantly higher risk for RRT (HR: 4.15, [95% CI: 3.27–5.27]), followed by those with HTN and DM (HR: 3.62, [95% CI: 2.82–4.65]), HTN and DLP (HR: 3.54, [95% CI: 2.91–4.31]), and HTN alone (HR: 3.10, [95% CI: 2.62–3.66]) compared with those without any three comorbidities. There was not a significant association between the risk of RRT and a drug history of metformin (HR: 0.68, [95% CI: 0.37, 1.25]) or NSAIDs use (HR: 0.75, [95% CI: 0.56, 1.00]).

**Fig. 4:**
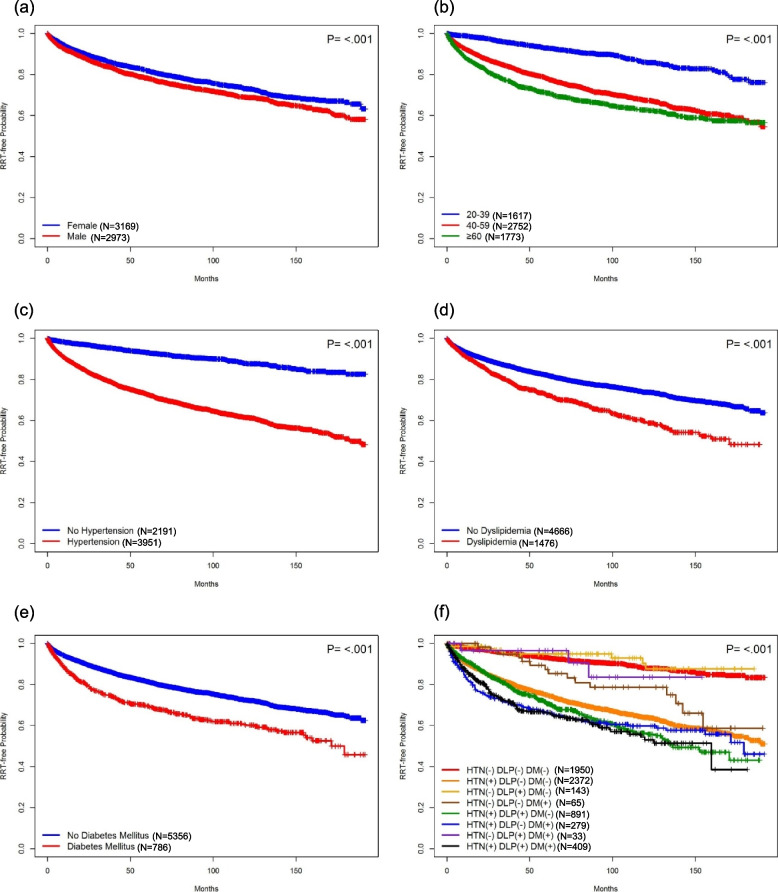
Kaplan–Meier curves for RRT-free probability for patients with ADPKD stratified by (**a**) gender (**b**) age (**c**-**e**) presence of comorbidity (**f**) combinations of HTN, DLP, and DM (*N* = number of patients in the corresponding subgroup). Kaplan–Meier curves depicted the renal replacement therapy (RRT)-free probability of patients with ADPKD and were compared between different subgroups using the log-rank test. Patients with ADPKD were stratified by gender, age, and individual and combined status of cardiometabolic comorbidities, including HTN, DLP, and DM

We executed Kaplan–Meier analysis of the RRT-free probabilities (Fig. 4). The RRT-free probabilities were remarkably lower in male sex, older age, presence of comorbid HTN, DM, DLP (all *p*-values < 0.001). Moreover, a cumulative effect was observed with increasing numbers of comorbidities of HTM, DM, and DLP (*p*-value < 0.001). And HTN was the most crucial risk factor among three comorbidities for poor RRT-free probability.

## Discussion

Our study illustrates for the first time that amalgamation of all cardiometabolic comorbidities significantly influences the risk of ESRD and all-cause mortality in the ADPKD population. We found that ADPKD patients with HTN and DM, HTN and DLP, or HTN alone had an increased mortality risk compared with those without cardiometabolic comorbidities (HTN, DM, DLP). Our study sheds light on the proactive management of cardiometabolic comorbidities, which may be beneficial for preventing early life entering ESRD and increasing overall survival in patients with ADPKD.

### HTN and ADPKD

According to the results shown in Figs. 2 and  3, HTN was recognized as the most important independent risk factor for renal and overall survival in this study, which is in accordance with previous publications [10, 22]. The HALT Progression of Polycystic Kidney Disease study concluded that strict blood pressure control with blockade of the renin-angiotensin system was associated with a slower kidney volume increase and greater reduction in the left ventricular mass index and urinary albumin excretion [23], shedding light on the blood pressure target of patients with ADPKD.

### DM and ADPKD

Our results suggest that DM is a significant risk factor for ESRD and overall survival. ADPKD is a well-known independent risk factor for new-onset diabetes after transplantation [24, 25]. Defective glucose metabolism and metabolic reprogramming have been reported in ADPKD models, which may indicate the possible harm of DM in ADPKD patients [26]. A retrospective cohort study compared pre-kidney transplant patients with both ADPKD and DM versus age- and sex-matched non-diabetic patients with ADPKD and observed that diabetic patients had a higher body mass index and greater kidney volume than those without DM [13]. In accordance with our study, the diabetic ADPKD patients had an earlier median age at onset of HTN compared to those with ADPKD alone. Moreover, diabetic ADPKD patients had an earlier median age at death compared to those with ADPKD alone. The prevalence of DM and metabolic syndrome is increasing in patients with ADPKD, similar to that in the general population. Cardiovascular disease, the most common cause of mortality and morbidity in patients with ADPKD, is associated with age, HTN, DM, DLP, and obesity [27]. These findings are consistent with our findings.

### DLP and ADPKD

Besides amino acid and glucose metabolism, fatty acid metabolism was also identified as a metabolic difference between healthy controls and patients with ADPKD. Insulin resistance and DLP have also been identified as subclinical cardiovascular abnormalities that contribute to cardiometabolic risk in ADPKD [28]. Another study reported a high prevalence of cardiovascular risks and events in patients with ADPKD, which contributed to high mortality [29]. Patients with DLP present with high oxidative stress, endothelial dysfunction, inflammation, and immune responses, which may contribute to this high cardiovascular risk [30].

Although according to our findings DLP was not an independent risk factor for renal survival and all-cause mortality, comorbid DLP and HTN with or without DM were noted to cause remarkable disease exacerbation in our study. A 3-year randomized controlled trial examining the effects of statins (HMG-CoA reductase inhibitors) in patients with ADPKD found that statins decreased the change in htTKV [30]. Although there are inconsistent findings regarding the benefits of statin therapy in slowing renal disease progression [31–33], reduced levels of low-density lipoprotein cholesterol (LDL-C) have been found to provide cardiovascular protection in ADPKD [34]. Owing to the lack of a clinical consensus regarding the guidelines for DLP management in patients with ADPKD, our study increases the awareness of active control and surveillance of these prevalent cardiometabolic comorbidities.

Moreover, our results were in accordance with the Consortium for Radiologic Imaging Studies of Polycystic Kidney Disease [35]. We confirmed that ADPKD was more severe in male than in female patients, and there was an elevated risk for ESRD and death with age, which could be explained by the age-dependent decrease in eGFR and increase in htTKV in the elderly [36, 37].

Given the high variability in renal function prognosis in patients with ADPKD, it is imperative to identify the risk factors that govern the rate of disease progression. We used RRT as the endpoint readout in renal function analysis in our renal dysfunction prediction model, which indicated a failure to compensate for kidney function by cyst proliferation, tubulointerstitial inflammation, and fibrosis [38]. Our data indicated that the risks for early renal function deterioration and entering ESRD displayed an accumulative relationship with an increase in the number of metabolic comorbidities, including HTN, DM, and DLP. Therefore, it is crucial for patients with ADPKD with metabolic comorbidities to take action to reduce risk factors, such as rigorous blood pressure control, lipid profile improvement, and blood glucose management.

Our data provide associations between cardiometabolic comorbidities and the risk for overall-mortality and RRT in ADPKD; however, our study has several limitations that require further investigation. First, the sample sizes of some subgroups were comparably small due to stratification by the combination of HTN, DLP, and DM, which lowered the statistical power of the study. In particular, the survival analyses of the HTN (-) DLP ( +) DM ( +), and metformin subgroups with a relatively small sample size may have resulted in a type II error. Second, owing to the claim data nature of the NHIRD, we defined the date of the first medical claim with the corresponding ICD-9-CM code of ADPKD as the date of diagnosis of ADPKD and the age at diagnosis. The real diagnosis date could lie before the 16-year study period and lead to errors in both ages at diagnosis and the follow-up period. Plus, documented renal outcome predictors for ADPKD patients, such as total kidney size, Mayo classification, and germline mutation, were not accounted for due to limited availability of clinical information in the claim data. In addition, although obesity is known to be independently associated with rate of progression in ADPKD [39], our claims-based analysis showed that there was remarkable undercoding of obesity in administrative claims data: only approximately 2% of patients in our dataset were diagnosed with obesity compared with an estimated 20% prevalence based on previous documentation [29, 39]. Although obesity is known to be independently associated with the rate of ADPKD progression [39], including obesity as a risk factor may lead to an underestimation of the number of obese patients with ADPKD due to limitations in the nature of the claims data, which may give rise to implausible inferences. Future studies are needed to discern the clinical burden of obesity and other cardiovascular factors in patients with ADPKD. In addition, our inclusion of medication use stems from documented evidence of NSAID-induced nephrotoxicity and the observed therapeutic benefits of metformin. We intended to account for the potential influence of medication history on the associations of cardiometabolic comorbidities in patients with ADPKD rather than leaving adjustments for medication out of our study. By incorporating the medication history, our study addressed potential confounding effects and provided a more comprehensive understanding of these associations. We recorded drug history regarding metformin and NSAIDs using a meaningful cutoff for clinically impactful consecutive usage while maintaining sensitivity for enhanced statistical power and insights into subsequent research. However, we acknowledge the lack of comprehensive evaluation of frequency, dose, and duration of drug use and a relatively small sample size of the subgroup with a positive drug history. Further investigations with more detailed medication data and a larger sample size would be valuable to delineate these associations. Finally, renal survival was defined as the time from disease onset to ESRD, which required RRT. Nonetheless, whether the first medical claim date was representative of disease onset might differ from case to case; therefore, the time intervals between diagnosis and initiation of RRT/death require cautious interpretation.

## Conclusion

The study presents the first time that cardiometabolic comorbidities (HTN, DM, and DLP) cumulatively increases the risk of RRT in patients with ADPKD who also had higher all-cause mortality. Thus, the findings reinforce the urgency of proactive prevention of HTN, DLP, and DM to improve renal outcomes and overall survival in ADPKD patients.

### Supplementary Information

**Additional file 1: Table S1.** Diagnosis criteria for ADPKD, HTN, DLP and DM. **Table S2.** Cross tabulation of baseline comorbidities of patient with ADPKD. **Table S3.** ATC Codes of NSAIDs. **Table S4.** Procedure codes of RRT. **Table S5. **Interaction test between RRT and comorbidities in overall survival analysis. **Figure S1.** Fine and Gray model for all-cause mortality accounting for competing risk with RRT. **Figure S2.** Kaplan–Meier curves for survival probability for patients with ADPKD stratified by (a) age (b) RRT status (c) combinations of HTN, DLP, and DM (N=number of patients in the corresponding subgroup). 

## Data Availability

The data that support the findings of this study are available from the Health and Welfare Data Science Center, Ministry of Health and Welfare, Taiwan (https://dep.mohw.gov.tw/DOS/cp-5119-59201-113.html), but restrictions apply to the availability of these data, which were under approval for the current study and so are not publicly available. The linked data set used in this study had to be applied from Ministry of Health and Welfare, Taiwan and analyzed in person in the Health and Welfare Data Science Center, Ministry of Health and Welfare, Taiwan. The processed data are however available from the corresponding author, Dr. Ta-Chien Chan upon reasonable request and with permission of the Health and Welfare Data Science Center, Ministry of Health and Welfare, Taiwan.

## Abbreviations

ADPKD: Autosomal-dominant polycystic kidney disease
RRT: Renal replacement therapy
HTN: Hypertension
DLP: Dyslipidemia
DM: Diabetes mellitus
PLD: Polycystic liver disease
HNIRD: National health insurance research database
HR: Hazard ratio
PC: Polycystin
eGFR: Estimated glomerular filtration rate
ESRD: End-stage renal disease
htTKV: Height-adjusted total kidney volume
HQOL: Health-related quality of life
PIN: Personal identification numbers
ATC code: Anatomical therapeutic chemical code
CI: Confidence interval
HALT-PKD: HALT progression of polycystic kidney disease
CRISP: Consortium for radiologic imaging studies of polycystic kidney disease
USRDS: United states renal data system
