# Supplementary material for: Cardiometabolic comorbidities in autosomal dominant polycystic kidney disease: a 16-year retrospective cohort study
Source: BMC Nephrol. 2023 Nov 9;24:333. doi: 10.1186/s12882-023-03382-0 (PMC10637020; doi:10.1186/s12882-023-03382-0)
Supplement: Supplementary file 1 — Additional file 1: Table S1. Diagnosis criteria for ADPKD, HTN, DLP and DM. Table S2. Cross tabulation of baseline comorbidities of patient with ADPKD. Table S3. ATC Codes of NSAIDs. Table S4. Procedure codes of RRT. Table S5. Interaction test between RRT and comorbidities in overall survival analysis. Figure S1. Fine and Gray model for all-cause mortality accounting for competing risk with RRT. Figure S2. Kaplan–Meier curves for survival probability for patients with ADPKD stratified by (a) age (b) RRT status (c) combinations of HTN, DLP, and DM (N=number of patients in the corresponding subgroup). [file 12882_2023_3382_MOESM1_ESM.pdf]

Supplementary Materials

Table S1: Diagnosis criteria for ADPKD, HTN, DLP and DM

Disease	Diagnosis Criteria
ADPKD	A diagnosis of ADPKD identified by ICD-9-CM: 753.13 in Taiwan's National Health Insurance Research Database (NHIRD) from 2000 to 2015
HTN	At least two diagnoses of HTN identified by ICD-9-CM codes 401-405 of outpatient visits within 365 calendar days or one diagnosis on admission before or at the time of ADPKD diagnosis in NHIRD from 2000 to 2015
DLP	At least two diagnoses of DLP identified by ICD-9-CM code 272 of outpatient visits within 365 calendar days or one diagnosis on admission before or at the time of ADPKD diagnosis in NHIRD from 2000 to 2015
DM	At least two diagnoses of DM identified by ICD-9-CM code 250 of outpatient visits within 365 calendar days or one diagnosis on admission before or at the time of ADPKD diagnosis in NHIRD from 2000 to 2015

Table S2: Cross tabulation of baseline comorbidities of patient with ADPKD

		HTN	
		+	-
DLP	+	1300	176
	-	2651	2015

P<0.001

		HTN	
		+	-
DM	+	688	98
	-	3263	2093

P<0.001

Table S3: ATC Codes of NSAIDs

Drug Name	ATC Code
ibuprofen	M01AE01
naproxen	M01AE02
ketoprofen	M01AE03
piroxicam	M01AC01
indomethacin	M01AB01
diclofenac	M01AB05
etodolac	M01AB08
nabumeton	M01AX01
meloxicam	M01AC06
celecoxib	M01AH01
etoricoxib	M01AH05

Table S4: Procedure codes of RRT

RRT modalities	Procedure code
Hemodialysis	58001C, 58019C, 58020C, 58021C, 58022C, 58023C, 58024C, 58025C, 58029C
Peritoneal dialysis	58002C, 58009A, 58009B, 58010A, 58010B, 58011A, 58011AB, 58011B, 58011C, 58012A, 58012B, 58017B, 58017C, 58026C, 58028C
Other dialysis	58018C, 58027C, 58030B
Renal transplantation	76020A, 76020B, 97416K, 97417A, 97418B

Table S5: Interaction test between RRT and comorbidities in overall survival analysis

Variables	RRT	
	P for Interaction	HR (95%CI)
HTN(+) DLP(-) DM(-)	<.001	0.54 (0.39, 0.75)
HTN(-) DLP(+) DM(-)	0.690	0.72 (0.15, 3.57)
HTN(-) DLP(-) DM(+)	0.533	0.74 (0.29, 1.90)
HTN(+) DLP(+) DM(-)	0.292	0.80 (0.54, 1.21)
HTN(+) DLP(-) DM(+)	<.001	0.44 (0.28, 0.69)
HTN(-) DLP(+) DM(+)	0.481	2.04 (0.28, 14.78)
HTN(+) DLP(+) DM(+)	0.012	0.57 (0.37, 0.88)

Figure S1: Fine and Gray model for all-cause mortality accounting for competing risk with RRT

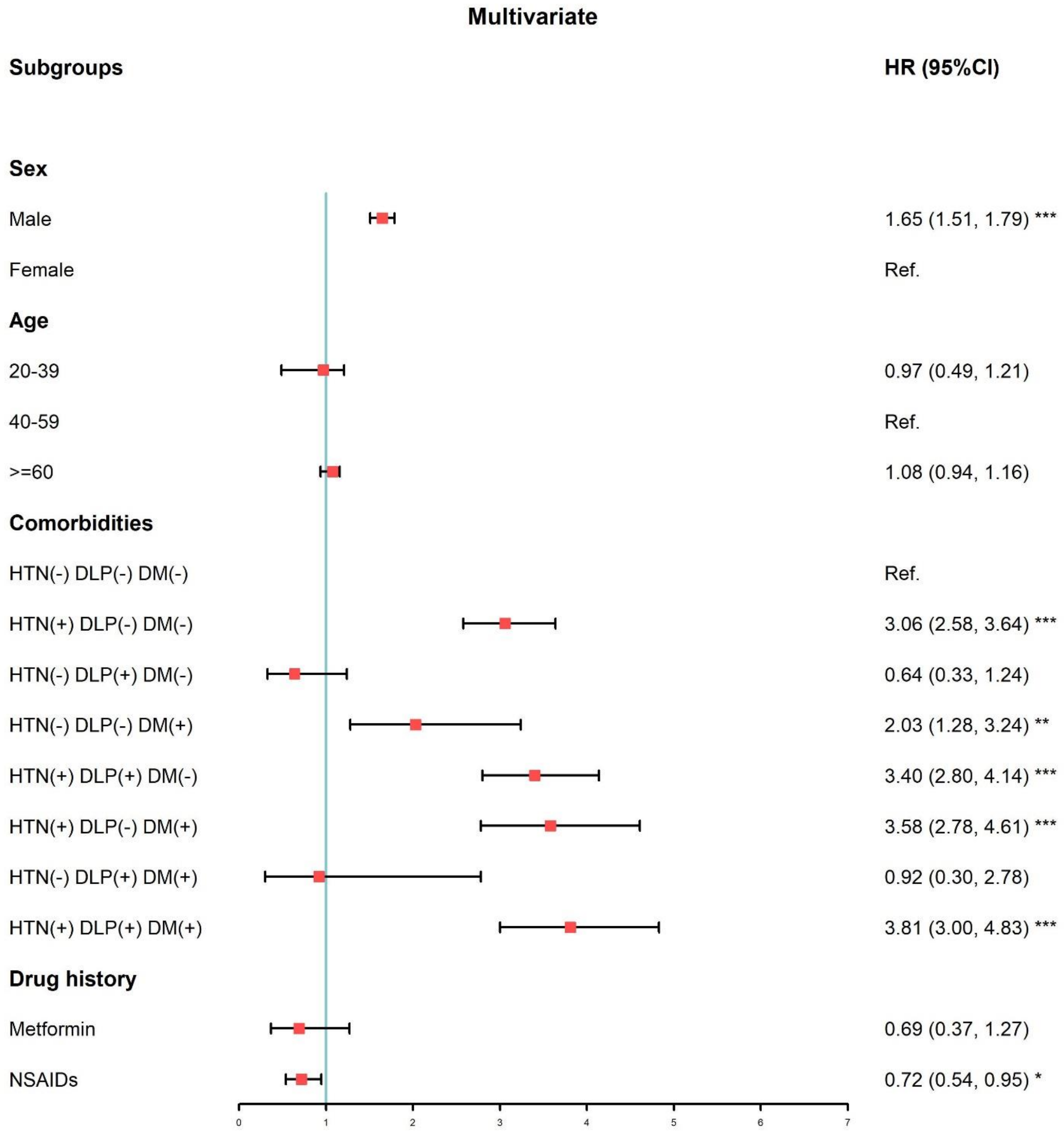

(*p<0.05, **p<0.01, ***p<0.001)

Figure S2: Kaplan–Meier curves for survival probability for patients with ADPKD stratified by (a) age (b) RRT status (c) combinations of HTN, DLP, and DM (N=number of patients in the corresponding subgroup)

(a)

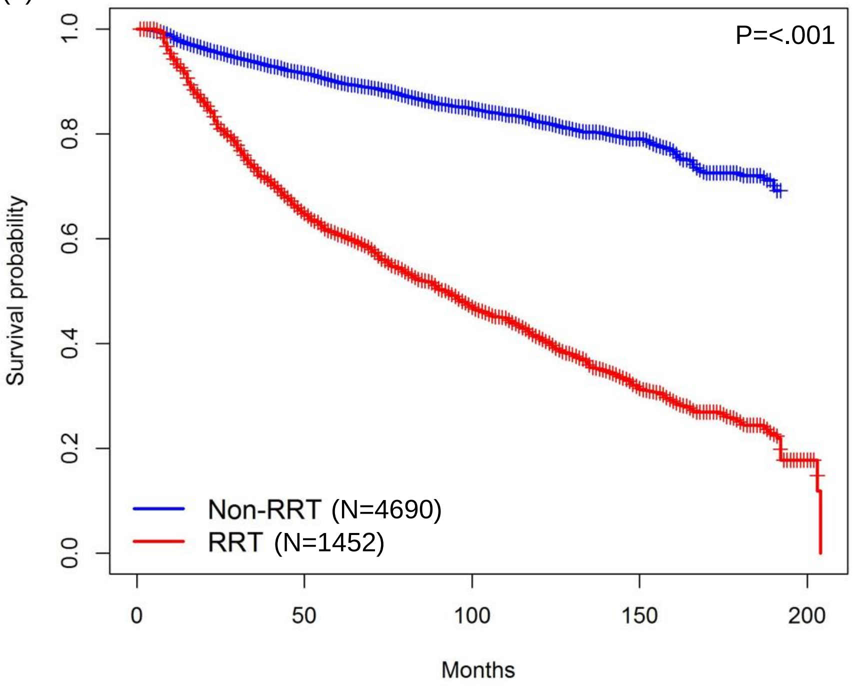

(b)

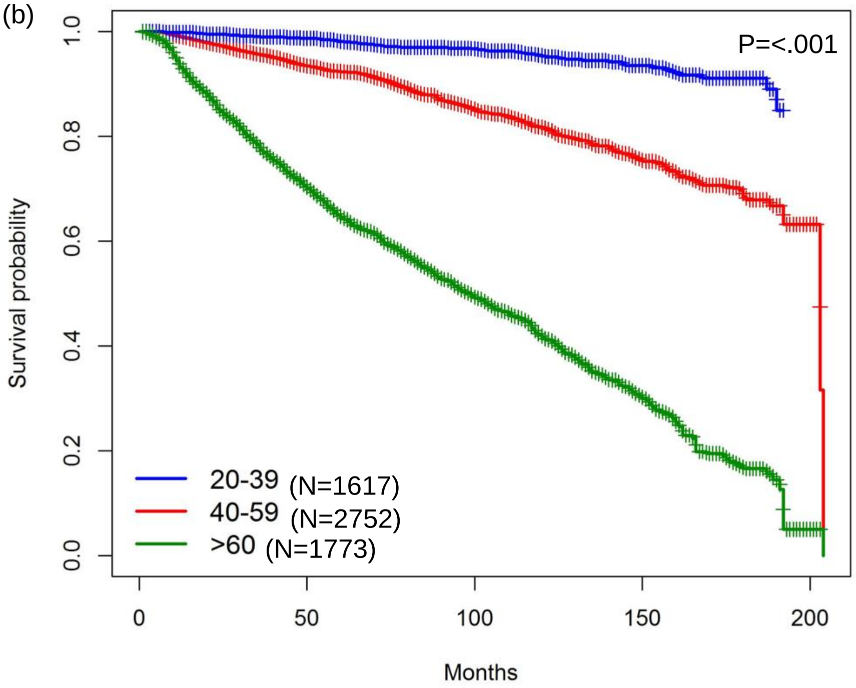

(c)

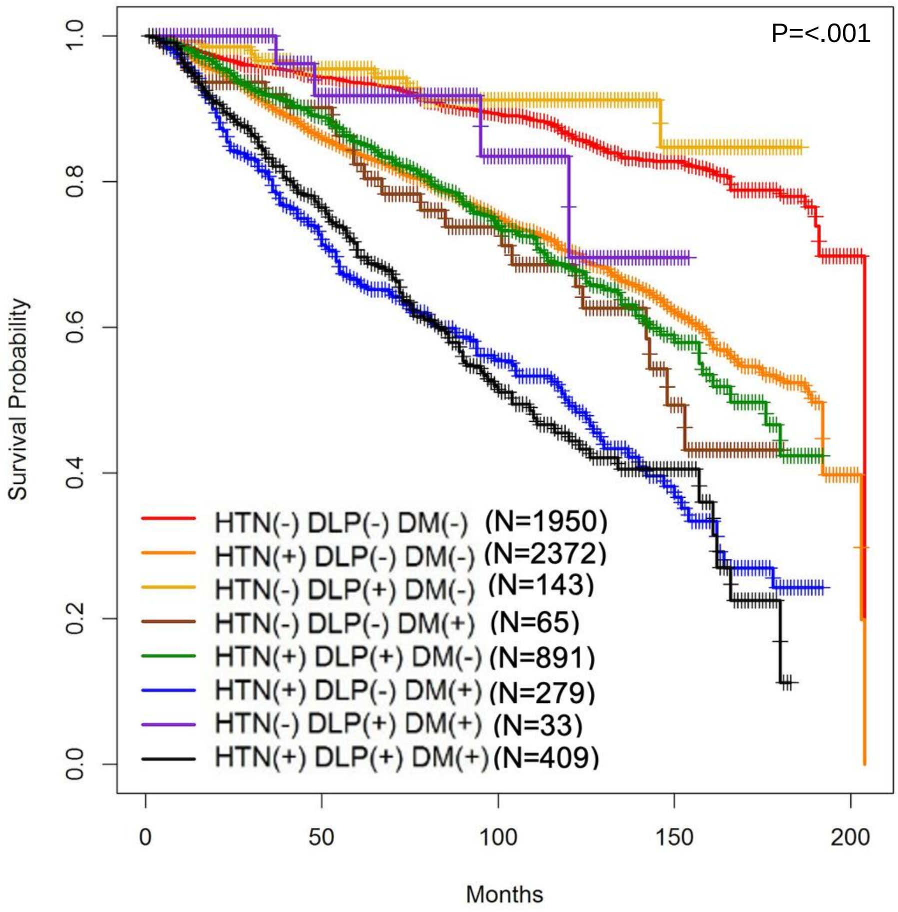
P=<.001
Survival Probability
1.0
0.8
0.6
0.4
0.2
0.0
HTN(-) DLP(-) DM(-) (N=1950)
HTN(+) DLP(-) DM(-) (N=2372)
HTN(-) DLP(+) DM(-) (N=143)
HTN(-) DLP(-) DM(+) (N=65)
HTN(+) DLP(+) DM(-) (N=891)
HTN(+) DLP(-) DM(+) (N=279)
HTN(-) DLP(+) DM(+) (N=33)
HTN(+) DLP(+) DM(+) (N=409)
0
50
100
150
200
Months
